# Comparison of maintenance with decitabine or chemotherapy in a real-world cohort of patients with acute myeloid leukemia 

**DOI:** 10.3389/fphar.2026.1753030

**Published:** 2026-02-13

**Authors:** Zhi-Feng Wei, Jia-Qi Yan, Ye-Hui Tan, Hai Lin, Qiu-Ju Liu, Xiao-Liang Liu, Long Su, Su-Jun Gao

**Affiliations:** 1 Department of Hematology, The First Hospital of Jilin University, Changchun, China; 2 Key Laboratory of Hematology Precision Medicine of Jilin Province, The First Hospital of Jilin University, Changchun, China

**Keywords:** acute myeloid leukemia, chemotherapy, decitabine, efficacy, maintenance therapy, safety

## Abstract

**Background:**

The objective of this real-world cohort study was to evaluate the efficacy and safety of decitabine versus conventional chemotherapy in the maintenance therapy (MT) of patients with acute myeloid leukemia (AML).

**Methods:**

Data were collected from 156 consecutive patients diagnosed with AML at our center. All patients achieved complete remission (CR) after 1–2 courses of induction therapy, followed by consolidation with high-dose cytarabine (HiDAC). MT was administered using either decitabine or conventional chemotherapy, while patients who did not receive maintenance served as controls.

**Results:**

MT significantly improved both relapse-free survival (RFS) and overall survival (OS) in AML patients. However, no significant difference was observed between decitabine and conventional chemotherapy. MT notably prolonged both RFS and OS in the cytogenetic intermediate-risk group, patients without *FLT3*-ITD mutations, and those achieving CR after one course of induction therapy. The benefits of MT were not influenced by the European Leukemia Net (ELN) risk category, measurable residual disease (MRD) status, or the number of HiDAC courses. Compared with chemotherapy, decitabine maintenance significantly improved RFS and OS in patients who received 3–4 courses of treatment. Additionally, the incidence of adverse reactions was significantly lower in the decitabine group than in the chemotherapy arm.

**Conclusion:**

MT with either decitabine or chemotherapy can improve outcomes of AML patients in this real-world cohort. Decitabine maintenance exhibits better tolerability compared with chemotherapy and enhances survival in specific patient subgroups.

## Introduction

Acute myeloid leukemia (AML) represents the most prevalent form of acute leukemia among adult patients, often associated with dismal prognoses. The latest epidemiological data indicate that approximately 22,010 new cases of AML are diagnosed each year, with 11,090 patients dying from this leukemia subtype in the United States (Siegel et al., 2025). In China, there are 38,571 new AML cases annually, and 20,613 patients succumb to the disease each year (Fu et al., 2025). Over the past decade, significant advancements have been made in improving AML outcomes, driven by the advent of novel targeted agents, cellular and molecular immunotherapies, optimized treatment strategies, and expanded access to hematopoietic stem cell transplantation (HSCT) (DiNardo et al., 2020; Erba et al., 2023; Lu et al., 2025; Zhang et al., 2021). However, the majority of patients still eventually experience disease relapse, and enduring survival remains suboptimal. Therefore, sustaining persistent remission and reducing the risk of relapse continue to be critical challenges. Maintenance therapy (MT) has emerged as a promising approach to achieve more durable remissions and enhance overall survival, yet its optimal application remains to be clearly established (Goulart et al., 2025; Senapati et al., 2023). The objective of MT is to eradicate residual leukemia cells, with treatment options encompassing chemotherapeutic agents, hypomethylating drugs, immunotherapies, and targeted therapies (Oran et al., 2020; Reville et al., 2021; Xuan et al., 2020). Repeated chemotherapy inevitably results in damage to normal tissues and cumulative toxicities. While decitabine and post-transplant low-dose azacitidine have been investigated as maintenance agents, neither has consistently shown a significant improvement in patient survival (Oran et al., 2020; Blum et al., 2017; Foran et al., 2019). The immune checkpoint inhibitors theoretically hold efficacy in inducing anti-leukemia effects. However, the precise role of these agents in AML MT remains unclear, especially considering the potential toxicities they may present (Reville et al., 2021; Liu et al., 2022). Molecular targeted therapy has also been investigated as a maintenance strategy for AML with specific genetic alterations. Posttransplant maintenance using the multikinase inhibitor sorafenib has been shown to improve both relapse-free survival (RFS) and overall survival (OS) in the context of allogeneic hematopoietic stem cell transplantation (allo-HSCT) (Xuan et al., 2020; Burchert et al., 2020). The first- and second-generation FLT3 inhibitors, midostaurin and gilteritinib, failed to yield additional improvements when used as MT in patients who underwent allo-HSCT (Maziarz et al., 2021; Levis et al., 2024). Notably, quizartinib may offer prominent benefit as post-remission maintenance for patients who did not receive consolidative allo-HSCT (Goulart et al., 2025). Trials evaluating IDH1/2 inhibitors, enasidenib and ivosidenib, in the posttransplant setting have demonstrated favorable tolerability and feasibility, with generally promising outcomes in terms of relapse and survival (Fathi et al., 2022; Fathi et al., 2023). Nevertheless, additional studies are still warranted.

China boasts a vast territory and faces imbalances in economic development and certain regional disparities in its healthcare system (Chen and Jin, 2022). These factors lead to poor accessibility to newly emerged therapeutic drugs or render them unaffordable due to economic constraints in a substantial proportion of patients with AML. Therefore, exploring ways to optimize currently accessible treatment approaches and generate more research evidence holds significant guiding value for improving clinical patient management and enhancing long-term prognosis. Randomized controlled trials (RCTs) are regarded as the “gold standard” for evaluating therapeutic efficacy. However, their results are constrained by strict inclusion and exclusion criteria. In contrast, real-world studies (RWSs), by reflecting actual clinical practice, enhance the practicality and generalizability of research findings, and they hold irreplaceable advantages particularly in personalized medicine, long-term efficacy assessment, and public health decision-making (Dang, 2023). Furthermore, the doses of consolidation therapy are relatively insufficient due to constitutional factors Chinese populations, medical conditions, and the incidence of comorbidities of patients with AML in many centers of China. Thus, the present study evaluates decitabine or conventional chemotherapy in MT in a real-world cohort of patients with AML. The results of this study may provide beneficial references for management of AML patients.

## Patients and methods

### Patients

Data were collected from 156 consecutive patients diagnosed with AML (excluding acute promyelocytic leukemia) ineligible for allo-HSCT between January 1, 2015, to December 21, 2023 in our center. The diagnosis of the patients was based on morphology, immunology, cytogenetics, and molecular biology (MICM). Standard culturing methods and chromosome-banding techniques were employed to analyze the karyotypes. Clonal abnormalities were identified and characterized in accordance with the International System for Human Cytogenetic Nomenclature (Shaffer et al., 2013). Potential molecular mutations of these patients were screened with a high-sensitivity next-generation sequencing approach, following the protocol detailed in our previous study (Su et al., 2018). The risk stratification of these patients was performed in accordance with the 2022 European Leukemia Net (ELN) risk classification system, which integrates both cytogenetic and molecular mutation data and is widely endorsed by hematologists and researchers (Döhner et al., 2022). All the patients provided informed consent prior to enrollment in the study. This study was approved by the Ethics Committee of the First Hospital of Jilin University (No. 2025-289) and conducted in accordance with the Declaration of Helsinki.

### Treatment

The majority of patients (94.9%, 148/156) received the standard “3 + 7” regimens (DA: daunorubicin plus cytarabine; IA: idarubicin plus cytarabine) for induction therapy. For patients over 60 years with poor physical status or complications, less intensive regimens such as DCAG (decitabine + cytarabine + aclarubicin + G-CSF), HAA (homoharringtonine + cytarabine + aclarubicin), or AA (cytarabine + aclarubicin) were adopted as well. All patients achieved complete remission (CR) after 1–2 induction therapy and proceeded to consolidation therapy with high-dose cytarabine (HiDAC; 1.5–3.0 g/m^2^) for 3–4 scheduled courses. However, alternative chemotherapeutic regimens (e.g., intermediate-dose cytarabine at 1.0–1.5 g/m^2^, DA, and DCAG) were administered to patients who developed severe toxic effects or infection complications during HiDAC-based consolidation. MT consisted of decitabine (20 mg/m^2^ for 5 days) or chemotherapy with homoharringtonine + cytarabine (HA), DA, or cytarabine + AA regimens administered for 2–4 courses at intervals of 6–8 weeks. Patients who did not receive MT served as the control group.

### Adverse reactions

Adverse reactions were evaluated and graded in accordance with the Common Terminology Criteria for Adverse Events (Version 5.0). During MT, the incidence and severity of various treatment-related adverse reactions in patients were recorded, including hematological toxicity (leukopenia, neutropenia, anemia, thrombocytopenia), gastrointestinal adverse reactions (nausea, vomiting), and infections (fever due to agranulocytosis, perianal infection, upper respiratory tract infection, pulmonary infection, etc.).

### Statistical analyses

Statistical Package for the Social Sciences (SPSS) software (Version 20.0) or Prism 10.1.2 (GraphPad Software) was used to calculate the statistical difference. OS was defined as the time from diagnosis to death from any cause or last follow-up. RFS was defined as the time from CR to death, relapse, or last follow-up. Unpaired t-tests or Mann-Whitney tests were used to compare two groups of continuous variables. One-way analysis of variance (ANOVA) was used to compare among groups with Dunnett’s multiple comparisons tests as *post hoc* analyses for three groups. The chi-square test or Fisher’s exact test was used to assess the statistical significance of differences between groups for categorical variables. The Kaplan-Meier method was employed for survival analysis, and the log-rank test was used to compare the differences between groups. Cox proportional hazard models were used to explore the factors affecting OS and RFS. Variables with *P* < 0.20 in univariable analysis were included in the multivariable analysis to identify the independent prognostic factors. A *P* < 0.05 was considered significant.

## Results

### Characteristics of the patients

Between 2015 and 2023, 156 patients with AML were included in the present study (Supplementary Table S1). The patient cohort comprised 78 males and 78 females. At diagnosis, the median age of the patients was 44 years (range: 15–63), with 149 patients (95.5%, 149/156) being younger than 60 years. Successful karyotype analyses were obtained in 123 cases (78.8%, 123/156). After screening by next-generation sequencing, at least one molecular mutation was identified in 152 patients (97.4%, 152/156), the top three mutated genes were *NPM1* (32.9%, 50/152), *FLT3*-ITD (25.0%, 38/152), and *DNMT3A* (23.7%, 36/152). According to the 2022 ELN risk category, 90 cases were classified as low-risk, 20 as intermediate-risk, and 13 as high-risk. The remaining 33 patients could not be stratified due to the lack of karyotype results. For initial induction therapy, 129 patients (82.7%, 129/156) received IA, 19 (12.2%, 19/156) received DA, and 8 (5.1%, 8/156) were treated with other regimens. In total, 144 patients (92.3%, 144/156) achieved composite complete remission (CRc) after one course of induction therapy, among whom 85 cases (59.0%, 85/156) showed negative measurable residual disease (MRD). This proportion increased to 116 patients (74.4%, 116/156) after the second course of therapy. All patients received consolidation therapy with HiDAC after achieving remission, with a median of 3 courses (range: 1–4). For MT, 55 patients received decitabine, 55 were treated with chemotherapy, and 46 patients did not receive any maintenance treatment.

### Long-term survival of the patients

**FIGURE 1:**
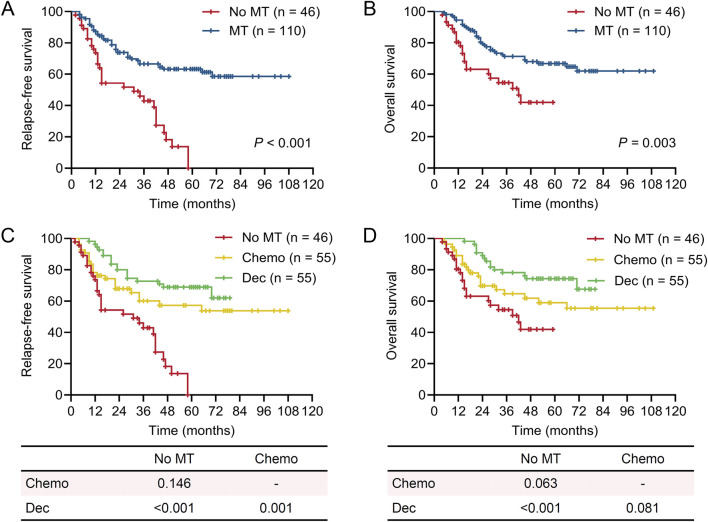
Survival of patients in different maintenance therapy groups. Kaplan-Meier plots showing relapse-free survival (RFS) and overall survival (OS) in different maintenance therapy (MT) groups. **(A)** RFS of patients who received MT or not. **(B)** OS of patients who received MT or not. **(C)** RFS of patients who received MT with decitabine (Dec), chemotherapy (Chemo), or not. **(D)** OS of patients who received MT with decitabine (Dec), chemotherapy (Chemo), or not. **(A‐D)** log-rank test.

After a median follow-up period of 43.5 months, 42 patients experienced relapse and 56 cases died. The 1-year, 3-year, and 5-year RFS was 93.6%, 80.0%, and 68.3%, respectively, while the corresponding OS was 93.6%, 82.4%, and 70.3% (Supplementary Figure S1A,B). We firstly evaluated the impact of MT on prognosis of the patients. The baseline features of these patients showed no significant difference suggesting comparability among the three groups (Table 1). As expected, MT significantly improved the outcomes of patients with AML in terms of both RFS and OS (Figure 1A,B). Moreover, both decitabine and conventional chemotherapy could remarkably reduce the risk of relapse and improve the survival of patients compared with no maintenance, but there is no obvious difference between decitabine and conventional chemotherapy (Figure 1C,D). Clonal characteristics of leukemia cells, responses to therapy, and treatment regimens all affect the outcomes of AML. Thus, univariable and multivariable analyses were employed to assess the potential elements that associated with prognoses of patients (Tables 2, 3). The results showed that MRD status after the second chemotherapy, numbers of HiDAC, and MT were the independent risk factors for RFS, whereas numbers of HiDAC and MT were the independent risk factors for OS. Taken together, benefit of MT with conventional chemotherapy or hypomethylation agents could be observed in this real-world AML patients cohort.

**TABLE 1:** Characteristics of AML patients in different groups.

Parameters	Decitabine (n = 55)	Chemotherapy (n = 55)	No MT (n = 46)	*P* values
Age	45 (17–63)	44 (15–58)	40 (15–63)	0.354
Gender	​	​	​	0.087
Males	23 (41.8%)	34 (61.8%)	21 (45.7%)	​
Females	32 (58.2%)	21 (38.2%)	25 (54.3%)	​
Cytogenetics	​	​	​	0.111
Low	21 (42.9%)	13 (41.9%)	9 (20.9%)	​
Intermediate	27 (55.1%)	17 (54.8%)	34 (79.1%)	​
High	1 (2.0%)	1 (3.2%)	0 (0.0%)	​
Genetic mutations				
*NPM1* mutation	21 (42.9%)	13 (41.9%)	9 (20.9%)	0.540
*FLT3*-ITD mutation	8 (14.5%)	11 (20%)	6 (13.0%)	0.595
*CEBPA* bzip mutation	6 (10.9%)	12 (21.8%)	11 (23.9%)	0.184
MDS-related	12 (21.8%)	8 (14.5%)	9 (19.6%)	0.606
ELN risk category (2022)	​	​	​	0.007
Low	41 (74.5%)	24 (43.6%)	25 (54.3%)	​
Intermediate	3 (5.5%)	5 (9.1%)	12 (26.1%)	​
High	5 (9.1%)	2 (3.6%)	6 (13.0%)	​
Unclassified	6 (10.9%)	24 (43.6%)	3 (6.5%)	​
Courses for CR	​	​	​	0.804
One	50 (90.9%)	52 (94.5%)	42 (91.5%)	​
Two	5 (9.1%)	3 (5.5%)	4 (8.7%)	​
MRD after couse one				
Negative	9 (20.9%)	13 (41.9%)	21 (42.9%)	​
Positive	34 (79.1%)	17 (54.8%)	27 (55.1%)	​
MRD after couse two	​	​	​	0.147
Negative	34 (61.8%)	43 (78.2%)	12 (26.1%)	​
Positive	21 (38.2%)	12 (21.8%)	34 (73.9%)	​
Courses of HiDAC	3 (1–4)	3 (1–4)	3 (1–4)	0.188

MT: maintenance therapy; MDS: myeloplastic syndrome; ELN: european leukemia net; CR: complete remission; MRD: measurable residual disease; HiDAC: high-dose cytarabine.

**TABLE 2:** Univariable analysis for relapse-free survival and overall survival.

Parameters	Relapse-free survival		Overall survival	
	HR (95% CI)	*P* values	HR (95% CI)	*P* values
High white blood cells	0.577 (0.249–1.335)	0.199	0.759 (0.274–2.101)	0.595
Cytogenetic groups	0.500 (0.268–0.929)	0.028	0.476 (0.235–0.964)	0.039
ELN risk category (2022)	​	<0.001	​	0.001
Low	1.000	​	1.000	​
Intermediate	0.356 (0.169–0.747)	0.006	0.362 (0.156–0.842)	0.018
High	1.172 (0.517–2.657)	0.703	1.217 (0.485–3.055)	0.675
*NPM1* mutation	1.143 (0.692–1.888)	0.602	1.009 (0.583–1.746)	0.975
*FLT3*-ITD mutation	0.465 (0.273–0.793)	0.005	0.427 (0.240–0.760)	0.004
*CEBPA* bzip mutation	1.153 (0.643–2.068)	0.634	1.509 (0.741–3.074)	0.257
MDS-related	1.150 (0.618–2.138)	0.660	1.262 (0.620–2.571)	0.521
CR after one course	2.714 (1.344–5.481)	0.005	2.718 (1.284–5.756)	0.009
MRD after couse one	0.781 (0.491–1.243)	0.298	0.784 (0.468–1.313)	0.355
MRD after couse two	0.614 (0.381–0.990)	0.046	0.669 (0.391–1.143)	0.141
Courses of HiDAC	2.412 (1.472–3.952)	<0.001	2.515 (1.466–4.314)	0.001
Maintenance therapy	3.083 (1.907–4.984)	<0.001	2.349 (1.363–4.036)	0.002

HR: hazard ratio; CI: confidence interval; MDS: myeloplastic syndrome; ELN: european leukemia net; CR: complete remission; MRD: measurable residual disease; HiDAC: high-dose cytarabine.

**TABLE 3:** Multivariable analysis for relapse-free survival and overall survival.

Parameters	Relapse-free survival		Overall survival	
	HR (95% CI)	*P* values	HR (95% CI)	*P* values
Cytogenetic groups	0.704 (0.324–1.527)	0.374	0.710 (0.292–1.727)	0.450
ELN risk category (2022)	​	0.570	​	0.626
Low	1.000	​	1.000	​
Intermediate	0.768 (0.293–2.009)	0.590	0.611 (0.217–1.722)	0.351
High	1.166 (0.454–2.998)	0.750	0.811 (0.288–2.286)	0.692
*FLT3*-ITD mutation	0.738 (0.332–1.640)	0.456	0.509 (0.217–1.192)	0.120
CR after one course	2.377 (0.925–6.106)	0.072	1.368 (0.049–3.820)	0.550
MRD after couse two	0.491 (0.264–0.912)	0.024	0.592 (0.283–1.239)	0.164
Courses of HiDAC	3.852 (2.139–6.937)	<0.001	3.631 (1.899–6.940)	<0.001
Maintenance therapy	4.579 (2.540–8.255)	<0.001	2.750 (1.421–5.321)	0.003

HR: hazard ratio; CI: confidence interval; ELN: european leukemia net; CR: complete remission; MRD: measurable residual disease; HiDAC: high-dose cytarabine.

### Potential influencing factors on the efficacy of MT

**FIGURE 2:**
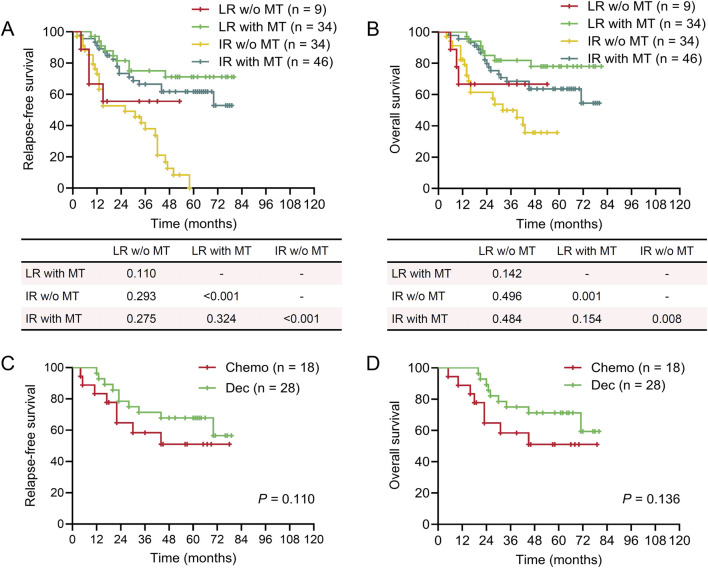
Survival of patients in different maintenance therapy groups based on cytogenetic risk category. Kaplan-Meier plots showing relapse-free survival (RFS) and overall survival (OS) in different maintenance therapy (MT) groups based on cytogenetic risk category. **(A)** RFS of patients who received MT or not in low- and intermediate-risk groups. **(B)** OS of patients who received MT or not in low- and intermediate-risk groups. **(C)** RFS of patients who received MT with decitabine (Dec) or chemotherapy (Chemo) in the intermediate-risk group. **(D)** OS of patients who received MT with decitabine (Dec) or chemotherapy (Chemo) in the intermediate-risk group. **(A‐D)** log-rank test.

**FIGURE 3:**
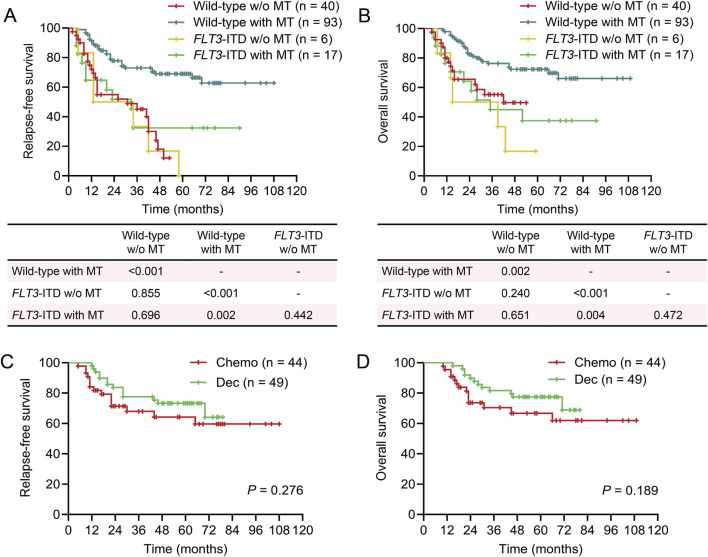
Survival of patients in different maintenance therapy groups based on *FLT3*-ITD mutations. Kaplan-Meier plots showing relapse-free survival (RFS) and overall survival (OS) in different maintenance therapy (MT) groups based on *FLT3*-ITD mutations. **(A)** RFS of patients who received MT or not in those with wild-type *FLT3* gene or *FLT3*-ITD mutations. **(B)** OS of patients who received MT or not in those with wild-type *FLT3* gene or *FLT3*-ITD mutations. **(C)** RFS of patients who received MT with decitabine (Dec) or chemotherapy (Chemo) in those with wild-type *FLT3* gene. **(D)** OS of patients who received MT with decitabine (Dec) or chemotherapy (Chemo) in those with wild-type *FLT3* gene. **(A‐D)** log-rank test.

Cytogenetic risk stratification was closely associated with the prognosis of patients with AML, as shown in the univariable analysis (Table 2). Given the small number of patients classified as high-risk, we only analyzed the impact of MT on outcomes in the low- and intermediate-risk groups. MT significantly improved both RFS and OS in the intermediate-risk group, which was not observed in patients with low-risk cytogenetics (Figure 2A,B). However, no significant difference was noted between decitabine and conventional chemotherapy in patients with intermediate risk karyotypes (Figure 2C,D). Intriguingly, patients in the intermediate-risk group who received MT exhibited outcomes similar to those in the low-risk group who did not receive MT (Figure 2A,B). The *FLT3*-ITD mutations predict poor prognosis in patients with AML, which was also confirmed in this study (Table 2). MT failed to provide additional benefit to patients with the *FLT3*-ITD mutations. In contrast, among patients with the wild-type *FLT3* gene, MT was associated with favorable RFS and OS, although there was no significant difference between decitabine and conventional chemotherapy (Figure 3A–D). The ELN risk classification was able to predict outcomes in patients with AML (Table 2). MT improved prognosis in both low- and intermediate-risk groups (Supplementary Figure S2A,B). Similarly, comparable trends of survival were observed between patients in the intermediate-risk group with MT and those in the low-risk group without (Supplementary Figure S2C,D). These results suggest that patients with intermediate-risk groups defined by cytogenetic or the ELN classification and those without *FLT3*-ITD mutations could benefit from MT.

**FIGURE 4:**
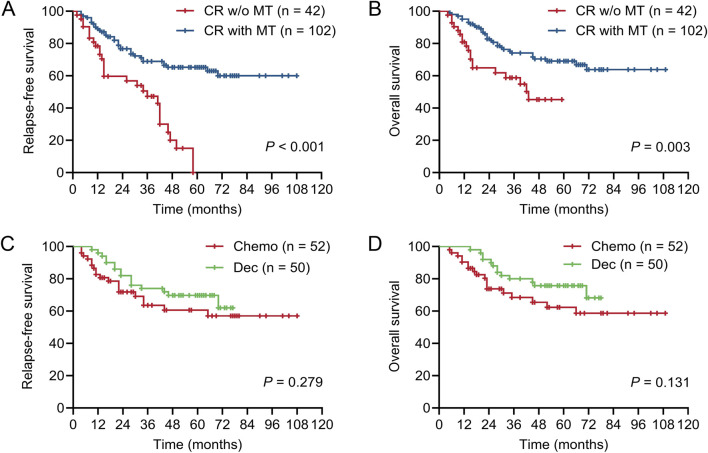
Impact of achieving CR on survival of patients in different maintenance therapy groups. Kaplan-Meier plots showing relapse-free survival (RFS) and overall survival (OS) in patients achieving CR after one course of induction therapy from different maintenance therapy (MT) groups. **(A)** RFS of patients who achieved CR in MT or no MT groups. **(B)** OS of patients who achieved CR in MT or no MT groups. **(C)** RFS of patients who achieved CR in decitabine (Dec) or chemotherapy (Chemo) groups. **(D)** OS of patients who achieved CR in decitabine (Dec) or chemotherapy (Chemo) groups. **(A‐D)** log-rank test.

**FIGURE 5:**
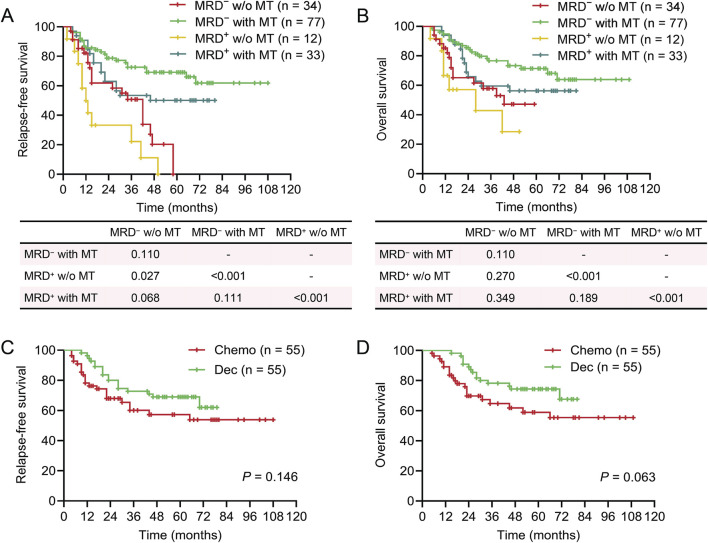
Survival of patients in different maintenance therapy groups based on MRD status. Kaplan-Meier plots showing relapse-free survival (RFS) and overall survival (OS) in different maintenance therapy (MT) groups based on MRD status after two courses of chemotherapy. **(A)** The influence of MRD status on RFS in patients who received MT or not. **(B)** The influence of MRD status on OS in patients who received MT or not. **(C)** RFS of patients with negative MRD who received MT with decitabine (Dec) or chemotherapy (Chemo). **(D)** OS of patients with negative MRD who received MT with decitabine (Dec) or chemotherapy (Chemo). **(A‐D)** log-rank test.

**FIGURE 6:**
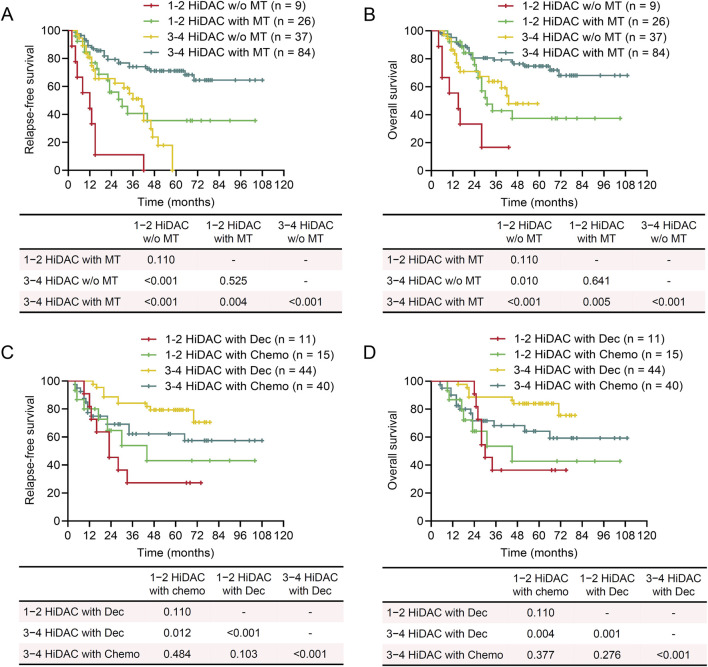
Impact of HiDAC courses on survival of patients in different maintenance therapy groups. Kaplan-Meier plots showing the influence of HiDAC courses on relapse-free survival (RFS) and overall survival (OS) in different maintenance therapy (MT) groups. **(A)** RFS of patients who received MT or not and were consolidated with 1–2 or 3–4 courses of HiDAC. **(B)** OS of patients who received MT or not and were consolidated with 1–2 or 3–4 courses of HiDAC. **(C)** RFS of patients who received MT with decitabine (Dec) or chemotherapy (Chemo) and were consolidated with 1–2 or 3–4 courses of HiDAC. **(D)** OS of patients who received MT with decitabine (Dec) or chemotherapy (Chemo) and were consolidated with 1–2 or 3–4 courses of HiDAC. **(A‐D)** log-rank test.

Patients who achieved CR after one course of chemotherapy exhibited favorable prognoses. These patients also derived benefits from MT in terms of both RFS and OS; however, there was no significant difference between decitabine and conventional chemotherapy (Figure 4A–D). Achieving MRD negativity after the second course of chemotherapy was associated with favorable RFS and OS. While MRD status did not influence the efficacy of MT, patients with positive MRD who received MT demonstrated comparable outcomes to those with negative MRD who did not receive MT (Figure 5A–D). This also suggests that MT may mitigate the adverse prognosis associated with positive MRD after two courses of chemotherapy. HiDAC is the primary consolidation regimen for patients ineligible for allo-HSCT. Patients consolidated with 3–4 courses of HiDAC had significantly superior RFS and OS compared to those treated with 1–2 courses. MT significantly improved prognosis regardless of the number of HiDAC courses administered (Figure 6A,B). Although no significant difference was observed between decitabine and conventional chemotherapy in patients consolidated with 1–2 courses of HiDAC, decitabine maintenance notably enhanced both RFS and OS in those treated with 3–4 courses (Figure 6A–D).

### Incidence of adverse reactions

We analyzed the incidence of adverse reactions between the decitabine and conventional chemotherapy groups (Table 4). The overall incidence of adverse reactions in the decitabine and conventional chemotherapy groups were 89.09% (49/55) and 94.55% (52/55), respectively. In the decitabine group, the most common hematological toxicities were leukopenia (76.4%), neutropenia (61.8%), and anemia (50.9%), while the most frequent non-hematological adverse events were liver dysfunction (34.5%), fever (20.0%), and pneumonia (9.1%). In the conventional chemotherapy group, the predominant hematological toxicities were leukopenia (78.2%), neutropenia (76.4%), and anemia (74.5%), whereas the most common non-hematological adverse events were fever (54.5%), liver toxicity (41.8%), and pneumonia (21.8%). The incidence and severity of hematological toxicities were significantly higher in the conventional chemotherapy group compared to the decitabine group. Specifically, the conventional chemotherapy group exhibited significantly higher incidences of grade 3–4 neutropenia (70.9% versus 34.5%, *P* < 0.001), grade 3–4 thrombocytopenia (70.9% vs. 20.0%, *P* < 0.001), and grade 3–4 anemia (43.6% vs. 7.3%, *P* < 0.001). The incidence of febrile neutropenia was also notably elevated in the conventional chemotherapy group (41.8% vs. 14.5%, *P* = 0.001). Regarding non-hematological toxicities, the conventional chemotherapy group had significantly higher rates of fever (54.5% vs. 20%, *P* < 0.001), nausea (10.9% vs. 0.0%, *P* = 0.012), vomiting (9.1% vs. 0.0%, *P* = 0.022), diarrhea (10.9% vs. 0.0%, *P* = 0.012), and rash (7.3% vs. 0.0%, *P* = 0.042) compared to the decitabine group.

**TABLE 4:** Frequencies of adverse reations in different groups.

​	Decitabine (n = 55)	Chemotherapy (n = 55)	*P* values
Hematological toxicities	Number (%)	Number (%)	
Neutropenia	34 (61.8%)	42 (76.4%)	0.099
Grade 3–4 neutropenia	19 (34.5%)	39 (70.9%)	<0.001
Thrombocytopenia	24 (43.6%)	40 (72.7%)	0.002
Grade 3–4 thrombocytopenia	11 (20.0%)	39 (70.9%)	<0.001
Anemia	28 (50.9%)	41 (74.5%)	0.010
Grade 3–4 anemia	4 (7.3%)	24 (43.6%)	<0.001
Leukopenia	42 (76.4%)	43 (78.2%)	0.820
Grade 3–4 leukopenia	19 (34.5%)	39 (70.9%)	<0.001
Febrile neutropenia	8 (14.5%)	23 (41.8%)	0.001
Non-hematological toxicities			
Fever	11 (20.0%)	30 (54.5%)	<0.001
Pneumonia	5 (9.1%)	12 (21.8%)	0.065
Hepatic toxicity	19 (34.5%)	23 (41.8%)	0.432
Renal toxicity	2 (3.6%)	0 (0.0%)	0.154
Nausea	0 (0.0%)	6 (10.9%)	0.012
Vomiting	0 (0.0%)	5 (9.1%)	0.022
Diarrhea	0 (0.0%)	6 (10.9%)	0.012
Constipation	1 (1.8%)	1 (1.8%)	1.000
Rash	0 (0.0%)	4 (7.3%)	0.042
Perianal infection	0 (0.0%)	2 (3.6%)	0.154
Upper respiratory tract infection	1 (1.8%)	3 (5.5%)	0.308

## Discussion

The present real-world cohort study evaluated the efficacy and safety of MT with decitabine versus conventional chemotherapy in patients with AML who had achieved remission after induction and consolidation therapy. The results demonstrate that MT significantly improves RFS and OS in this population, with decitabine demonstrating better tolerability and subgroup-specific survival benefits. These findings may provide valuable insights into optimizing maintenance strategies for AML patients in clinical practice.

Our data demonstrate that MT, whether with decitabine or conventional chemotherapy, consistently reduces relapse risk and improves survival compared to no maintenance, aligning with growing evidence supporting its role in post-remission care (Karakus et al., 2022; Senapati et al., 2022). This is particularly relevant in real-world settings, where patients often face barriers to intensive consolidation (e.g., dose-limiting toxicities, comorbidities, or limited access to high-dose regimens), making MT a critical bridge to sustained remission. Notably, the benefits of maintenance were observed across diverse patient subgroups, independent of ELN risk category, MRD status after two courses, or the number of HiDAC consolidation cycles. This universality underscores MT as a foundational strategy to consolidate remission, even in patients with suboptimal responses to initial therapy. A key finding of this study is the pronounced benefit of MT in cytogenetic or ELN intermediate-risk patients. This subgroup, which was associated with inferior outcomes compared to low-risk patients but without the aggressive trajectory of high-risk disease, often faces an “intermediate” prognosis that is difficult to optimize (Shimony et al., 2025). Our data show that MT narrows this gap: intermediate-risk patients receiving maintenance achieved survival outcomes comparable to low-risk patients without maintenance. This suggests that maintenance can effectively mitigate the adverse prognostic impact of intermediate-risk cytogenetics, offering a practical strategy to “upgrade” outcomes in this vulnerable population. Equally compelling is the observation that MT ameliorates the poor prognosis of MRD-positive patients after two courses of therapy. MRD positivity is a well-established marker of residual disease and a strong predictor of relapse (Heuser et al., 2021; van Weelderen et al., 2023). In our cohort, MRD-positive patients receiving maintenance achieved survival comparable to MRD-negative patients without maintenance, indicating that maintenance can suppress residual leukemia cells and “rescue” outcomes in those with persistent disease. This supports the use of maintenance as a targeted intervention for MRD-positive patients, particularly in settings where more intensive interventions (e.g., allo-HSCT) are not feasible. Intriguingly, decitabine showed distinct advantages in patients who received 3–4 cycles of HiDAC consolidation. The superiority of decitabine in this context may stem from its mechanism of direct anti-leukemia and immunomodulatory effects, which was reported to activate dendritic cells (Kwon et al., 2020), reinforce CD8^+^ T cell function (Liu et al., 2023), and promote the maintenance of effector function and memory phenotype of T cells (Kang et al., 2022). This profile makes it particularly well-suited for patients who have completed HiDAC consolidation, as it minimizes the risk of cumulative toxicity while sustaining anti-leukemic activity. It should be noted that the majority of patients (95.5%) in this study were younger than 60 years. Existing evidence has already shown that such a patient subgroup (aged <60 years) failed to benefit from decitabine maintenance (Blum et al., 2017). The difference may derive from relatively lower doses of HiDAC (not all patients received 3 g/m^2^ cytarabine) used in the present study due to constitutional factors, medical conditions, and the occurrence of comorbidities. For such patients, decitabine represents a tolerable and effective long-term maintenance option, reducing the burden of treatment-related complications that often limit adherence in real-world settings. Moreover, decitabine MT yields an OS of approximately 50.0%–68.0% among non-transplant AML patients (Blum et al., 2017; Liu et al., 2022), which is somewhat lower than the 78.2% survival rate observed in the present study. This discrepancy may stem from the distinct genetic backgrounds of the patient cohorts (with 7% vs. 2.0% of patients harboring high-risk cytogenetics in this study) and the limited sample size. Therefore, further investigations with larger patient cohorts are warranted to clarify this issue and validate the aforementioned findings.

Not all subgroups benefited equally from MT. Cytogenetic low-risk patients showed no significant improvement with maintenance, consistent with their favorable prognosis following standard consolidation (Shimony et al., 2025). This suggests that low-risk patients may be overtreated with maintenance, and resources could be redirected to higher-risk groups. Likewise, *FLT3*-ITD-mutated patients derived no benefit from either of the maintenance strategies evaluated here. *FLT3*-ITD mutations drive leukemogenesis and confer resistance to conventional therapy (Zalpoor et al., 2022; Tang et al., 2024). The ineffectiveness of decitabine or chemotherapy in this subgroup highlights the need for *FLT3* inhibitors (e.g., midostaurin, gilteritinib) as maintenance agents in *FLT3*-ITD-positive AML, a strategy supported by clinical trials showing reduced relapse risk with FLT3 inhibitor maintenance (Stone et al., 2017). Unfortunately, this study did not include these targeted agents, reflecting the limitations in accessibility to novel small-molecule drugs in real-world settings.

This study has several limitations. First, its retrospective design, small sample size, and certain variations in chemotherapy maintenance regimens may introduce selection bias, particularly in the non-random assignment of maintenance regimens. Second, the cohort lacks data on recently approved agents, such as FLT3 inhibitors and BCL-2 inhibitors (venetoclax), which have transformed AML therapy (Garcia et al., 2024). Integrating these agents into maintenance strategies, either alone or in combination with HMAs, warrants investigation in future prospective studies. Third, the homogeneous nature of a single-center cohort may limit generalizability, especially given regional disparities in AML management across China. Multi-center, prospective trials are needed to validate these findings in diverse populations. Finally, long-term follow-up beyond 5 years is required to assess the durability of maintenance benefits, particularly in certain subgroups.

In conclusion, this real-world study reinforces the value of MT in AML, with particular benefits in intermediate-risk and MRD-positive patients. Decitabine emerges as a superior option for patients who have completed adequate HiDAC consolidation, offering better tolerability and survival gains. For low-risk patients, maintenance may be unnecessary, while *FLT3*-ITD-mutated patients require alternative strategies, such as FLT3 inhibitors. These findings provide actionable insights to personalize MT, addressing unmet needs in resource-constrained settings and emphasizing the need for broader access to targeted agents in future practice.

## Data Availability

The original contributions presented in the study are included in the article/Supplementary Material, further inquiries can be directed to the corresponding authors.
