# Supplementary material for: Comparison of maintenance with decitabine or chemotherapy in a real-world cohort of patients with acute myeloid leukemia
Source: Front Pharmacol. 2026 Feb 13;17:1753030. doi: 10.3389/fphar.2026.1753030 (PMC12946668; doi:10.3389/fphar.2026.1753030)
Supplement: Supplementary file 1 [file DataSheet1.docx]

**Supplemental materials**

**Comparison of maintenance with decitabine or chemotherapy in a real-world cohort of patients with acute myeloid leukemia**

Zhi-Feng Wei ^a, b^, Jia-Qi Yan ^a, b^, Ye-Hui Tan ^a, b^, Hai Lin ^a, b^, Qiu-Ju Liu ^a, b^, Xiao-Liang Liu ^a, b^, Long Su ^a, b,^ *, Su-Jun Gao ^a, b,^ *

^a^ Department of Hematology, The First Hospital of Jilin University; Changchun 130021, China.

^b^ Key Laboratory of Hematology Precision Medicine of Jilin Province, The First Hospital of Jilin University; Changchun 130021, China.

*Corresponding author:

Long Su: Tel.: +86-0431-88782172; Fax: +86-0431-88786134; E-mail address: sulong@jlu.edu.cn. Department of Hematology, The First Hospital of Jilin University; No.1, Xinmin Avenue, Changchun 130021, Jilin Province, China.

Su-Jun Gao: Tel.: +86-0431-88782157; Fax: +86-0431-88786134; E-mail address: sjgao@jlu.edu.cn. Department of Hematology, The First Hospital of Jilin University; Changchun 130021, China.

Supplemental Table 1 Characteristics of AML patients enrolled in this study

| Parameters | Number or number (%) |
| --- | --- |
| Age | 44 (15−63) |
| Gender |  |
| Males | 78 (50.0%) |
| Females | 78 (50.0%) |
| Cytogenetics |  |
| Low | 43 (39.0%) |
| Intermediate | 78 (63.4%) |
| High | 2 (1.6%) |
| Genetic mutations |  |
| *NPM1* mutation | 50 (32.1%) |
| *FLT3*-ITD mutation | 38 (24.4%) |
| *CEBPA* bzip mutation | 29 (18.6%) |
| MDS-related | 29 (18.6%) |
| ELN risk category (2022) |  |
| Low | 90 (57.7%) |
| Intermediate | 20 (12.8%) |
| High | 13 (8.33%) |
| Unclassified | 33 (21.2%) |
| Courses for CR |  |
| One | 144 (92.3%) |
| Two | 12 (7.7%) |
| MRD after couse one |  |
| Negative | 85 (59.0%) |
| Positive | 59 (41.0%) |
| MRD after couse two |  |
| Negative | 116 (74.4%) |
| Positive | 40 (25.6%) |
| Courses of HiDAC | 3 (1−4) |
| Maintenance therapy |  |
| Yes | 110 (70.5%) |
| No | 46 (29.5%) |

MDS: myeloplastic syndrome; ELN: European Leukemia Net; CR: complete remission; MRD: measurable residual disease; HiDAC: high-dose cytarabine.


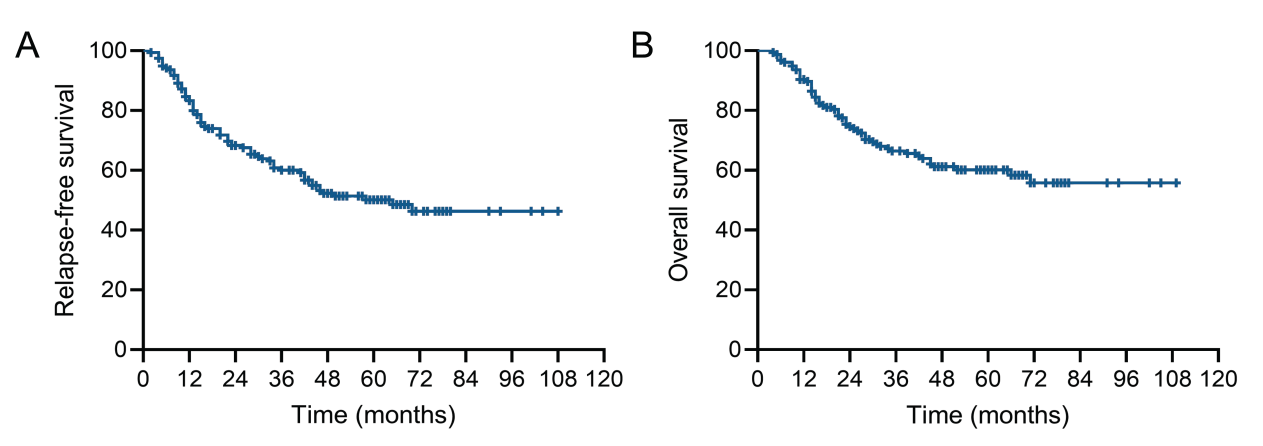


**Supplemental Figure 1 Survival of the whole cohort of patients enrolled in this study.**

Kaplan-Meier plots showing relapse-free survival (RFS; **A**) and overall survival (OS; **B**).


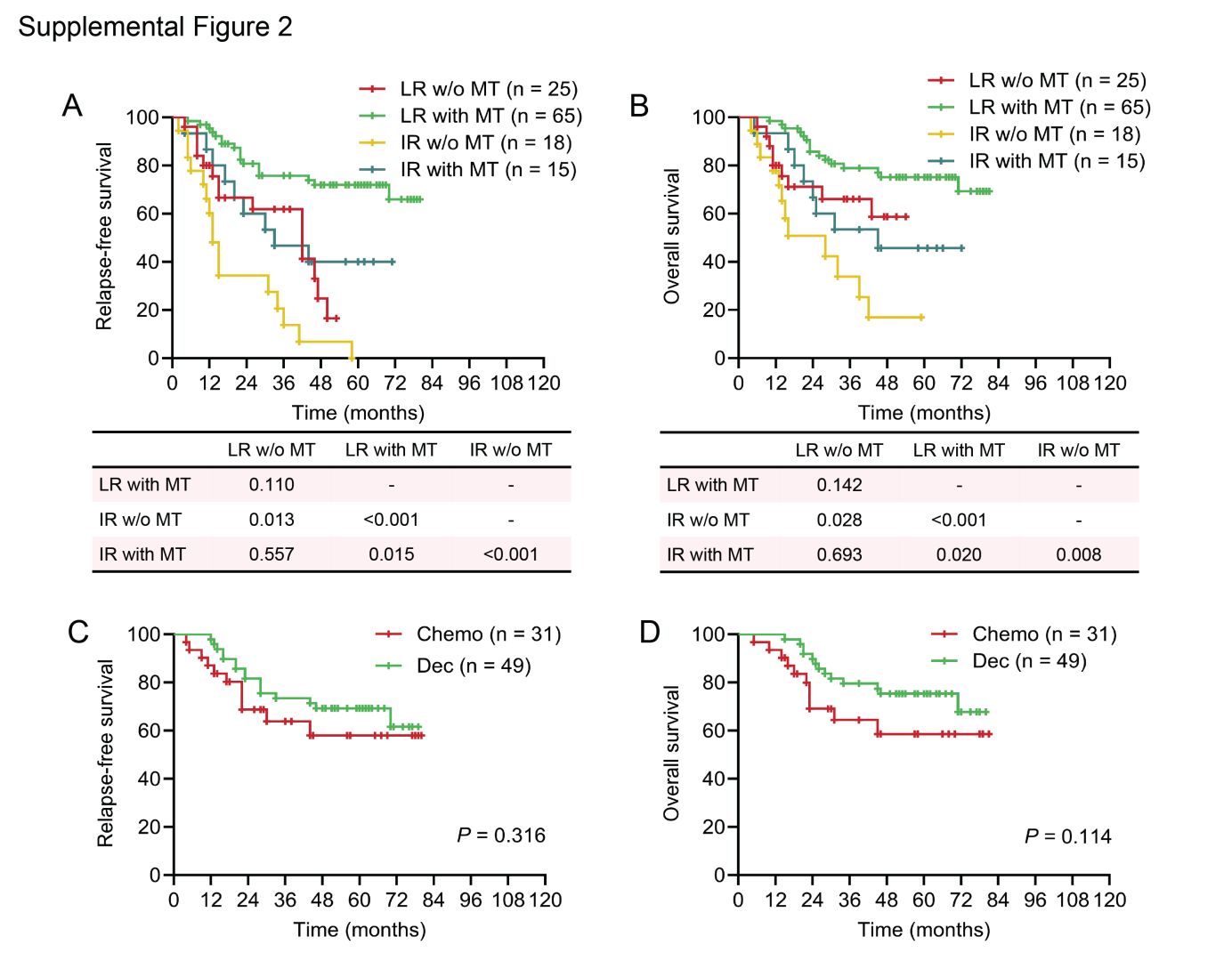


**Supplemental Figure 2 Survival of patients in different maintenance therapy groups based on ELN risk category.**

Kaplan-Meier plots showing relapse-free survival (RFS) and overall survival (OS) in different maintenance therapy (MT) groups based on ELN risk category. (**A**) RFS of patients who received MT or not in low- and intermediate-risk groups. (**B**) OS of patients who received MT or not in low- and intermediate-risk groups. (**C**) RFS of patients who received MT with decitabine (Dec) or chemotherapy (Chemo) in the intermediate-risk group. (**D**) OS of patients who received MT with decitabine (Dec) or chemotherapy (Chemo) in the intermediate-risk group. A–D: log-rank test.
